# The KdpD/KdpE Two-Component System: Integrating K^+^ Homeostasis and Virulence

**DOI:** 10.1371/journal.ppat.1003201

**Published:** 2013-03-28

**Authors:** Zoë N. Freeman, Steve Dorus, Nicholas R. Waterfield

**Affiliations:** 1 Department of Biology and Biochemistry, University of Bath, Bath, United Kingdom; 2 Department of Biology, Syracuse University, Syracuse, New York, United States of America; International Centre for Genetic Engineering and Biotechnology, India

## Abstract

The two-component system (TCS) KdpD/KdpE, extensively studied for its regulatory role in potassium (K^+^) transport, has more recently been identified as an adaptive regulator involved in the virulence and intracellular survival of pathogenic bacteria, including *Staphylococcus aureus*, entero-haemorrhagic *Escherichia coli*, *Salmonella typhimurium*, *Yersinia pestis*, *Francisella* species, *Photorhabdus asymbiotica*, and mycobacteria. Key homeostasis requirements monitored by KdpD/KdpE and other TCSs such as PhoP/PhoQ are critical to survival in the stressful conditions encountered by pathogens during host interactions. It follows these TCSs may therefore acquire adaptive roles in response to selective pressures associated with adopting a pathogenic lifestyle. Given the central role of K^+^ in virulence, we propose that KdpD/KdpE, as a regulator of a high-affinity K^+^ pump, has evolved virulence-related regulatory functions. In support of this hypothesis, we review the role of KdpD/KdpE in bacterial infection and summarize evidence that (i) KdpD/KdpE production is correlated with enhanced virulence and survival, (ii) KdpE regulates a range of virulence loci through direct promoter binding, and (iii) KdpD/KdpE regulation responds to virulence-related conditions including phagocytosis, exposure to microbicides, quorum sensing signals, and host hormones. Furthermore, antimicrobial stress, osmotic stress, and oxidative stress are associated with KdpD/KdpE activity, and the system's accessory components (which allow TCS fine-tuning or crosstalk) provide links to stress response pathways. KdpD/KdpE therefore appears to be an important adaptive TCS employed during host infection, promoting bacterial virulence and survival through mechanisms both related to and distinct from its conserved role in K^+^ regulation.

## Introduction

Two-component systems (TCSs) are widespread regulatory systems that enable microbes to control their cellular functions and respond appropriately to a diverse range of stimuli such as pH, osmolarity, quorum signals, or nutrient availability. The two components are a histidine kinase (HK), which senses an environmental signal, and a response regulator (RR), which mediates a cellular response, typically by altering expression of target genes. The genes *kdpD* and *kdpE* together encode the KdpD/KdpE TCS, which is well-studied for its regulation of the Kdp-ATPase potassium ion (K^+^) pump operon *kdpFABC*. Although best studied in *Escherichia coli*, highly homologous genes are found in most other bacteria, and are assumed to function similarly in K^+^ homeostasis. However, the signalling networks regulating basic bacterial physiology and bacterial virulence are sometimes intricately linked [1], and TCSs are no exception. A classic example of a TCS with pleiotropic roles in homeostasis and virulence is PhoP/PhoQ, whose regulon both mediates responses to Mg^2+^-limited environments and governs virulence and intra-macrophage survival in a range of Gram negative species [2], [3]. More recently, the GraS/GraR TCS of *Staphylococcus aureus* has also been shown to possess functional diversity—it was best known for controlling resistance to antimicrobial peptides, but further investigation has revealed links to quorum sensing, stress response, cell wall metabolism, and regulation of haemolytic and fibrinogen-binding proteins [4]. Here, we review evidence from diverse and clinically relevant bacterial species, indicating that the KdpD/KdpE TCS responds to pathogenesis-related signals, directly regulates virulence genes, and mediates stress resistance.

## KdpD/KdpE and the Regulation of K^+^ Homeostasis during Pathogenesis

### Regulation of K^+^ Homeostasis by KdpD/KdpE

K^+^ uptake in bacteria occurs via different combinations of nonspecific channels and specialised transport systems [5]. Constitutively expressed systems, such as Trk, satisfy general K^+^ requirements, but the Kdp-ATPase is a specialised, inducible higher-affinity K^+^ pump [6]. The Kdp-ATPase itself is encoded by the structural operon *kdpFABC*. The Kdp-ATPase is widespread throughout the prokaryotes and the KdpD/KdpE TCS is found in over 1,082 bacterial and archaeal species [7]. Expression of the operon is triggered by three stimuli perceived and integrated by the HK KdpD: K^+^ concentration, osmolarity, and ATP concentration [8]. Turgor pressure has also been suggested as a stimulus for KdpD/KdpE activation, although this has been a subject of debate [8]. Mutation analysis suggests that an activating stimulus causes the inhibition of the phospho-KdpE-specific phosphatase activity of KdpD, leading to an accumulation of phospho-KdpE. This in turn binds to an operator sequence in the promoter DNA to activate transcription of *kdpFABC* [9]. Its regulation by a TCS makes Kdp the only known bacterial K^+^ transport system whose expression is strongly controlled at the level of transcription [5].

### Regulation of K^+^ Homeostasis during Pathogenesis

K^+^ is the single most abundant ion in the intracellular environment and its regulation is crucial for maintenance of cell turgor and for diverse processes contributing to normal homeostasis [5]. Many studies have shown that K^+^ regulation is critical for bacterial virulence [10]–[13]. Similarly, it is an intricate aspect of host response to pathogens; in neutrophils, the active transport of K^+^ across the phagosomal membrane releases antimicrobial peptides, activating proteases and enabling Reactive Oxygen Species (ROS)–mediated killing of engulfed bacteria [14]. Thus, K^+^ sequestration from a common limited supply may be critical to the competition between the bacterium and host. Considering the importance of K^+^ regulation in virulence, it may not be surprising that genes involved in K^+^ regulation and transport are critical to the survival of pathogenic bacteria. Transcriptomic evidence reveals that transcription of the *kdpD* gene is repressed at least 3-fold and in some cases up to 20-fold in *Staphylococcus aureus* in response to phagocytosis by human neutrophils (after 3 h of interaction) [15], and up-regulated in *Mycobacterium avium* during early growth in human macrophages (at 48 h after infection) [16].

## Role of KdpD/KdpE for Virulence and Pathogen Survival

### KdpD/KdpE Aids Bacterial Survival by Increasing K^+^ Uptake

Although the precise mechanisms of action are yet to be determined, infection models suggest that KdpD/KdpE increases the ability of some bacteria to cause disease or to survive within a host cell or animal (Table 1). For example, increased transcription of *kdpD/kdpE* was concurrent with increased survival of *S. aureus* in macrophages and deletion of the TCS resulted in attenuated survival [17], [18]. In human neutrophils, a *Y. pestis kdpD/kdpE* mutant was more readily killed compared to the wild-type strain [3]. A study that aimed to identify genes involved in persistent infection of the *Caenorhabditis elegans* intestine highlighted the impact of *Salmonella typhimurium kdpD* [19]. Further characterisation revealed that *kdpD* was in fact required for *S. typhimurium* infection of the nematode and also for survival in macrophage cell lines. In the nematode feeding assays, worms that had grazed on the *kdpD* mutant strain lived significantly longer than those fed on the wild-type bacteria, and in macrophages, intracellular growth and cytotoxicity were reduced [19]. These observations from intracellular models are generally consistent with the importance of K^+^ scavenging in K^+^ limited environments, as would be found in a phagosomal vacuole.

**Table 1:** Evidence supporting the role of KdpD/KdpE in bacterial virulence and survival in cell or animal models.

Bacterium	Model	Link to Virulence/Survival	References
*Staphylococcus aureus*	Human blood	Knock-out (KO) decreases survival	Xue et al., 2011 [17]
	Human blood	Increased transcription of *kdpD/kdpE* is concurrent with increased survival in blood; KO is attenuated.	Zhao et al., 2010 [18]
	Macrophage	KO decreases survival	Xue et al., 2011 [17]
	Macrophage	Increased transcription of *kdpD/kdpE* is concurrent with increased survival in blood; KO is attenuated	Zhao et al., 2010 [18]
	Neutrophil	*kdpD* is differentially transcribed during phagocytosis	Voyich et al., 2005 [15]
*Salmonella typhimurium*	Nematode	KpdD/KdpE is required for colonisation of the worm; KO is attenuated	Alegado et al., 2011 [19]
	Macrophage	KO decreases survival	Alegado et al., 2011 [19]
*Mycobacterium tuberculosis*	Mouse	KO increases virulence	Parish et al., 2003 [42]
*Mycobacterium avium*	Macrophage	*kdpD* is differentially transcribed during phagocytosis	Hou et al., 2002 [16]
*Yersinia pestis*	Neutrophil	KO decreases survival	O'Loughlin et al., 2010 [3]
*Photorhabdus asymbiotica*	Insect phagocyte	Transgenic expression of *P. asymbiotica* KdpD/KdpE in *Escherichia coli* increases intracellular survival	Vlisidou et al., 2010 [38]
*Francisella novicida*	Fruit fly	Mutation of *kdpD*, *kdpE*, *kdpA*, or *kdpC* is attenuating in a competitive index assay	Moule et al., 2010 [47]
*Francisella tularensis*	Mouse	Mutation of KdpD is attenuating for growth and survival	Weiss et al., 2007 [48]
Enterohaemorrhagic *Escherichia coli* (EHEC)	HeLa cells	Deletion of *kdpE* resulted in slightly fewer lesion “pedestals" in infected cells; double deletion of *kdpE* and *cra* prevented pedestal formation	Njoroge et al., 2012 [25]

### Other KdpD/KdpE Mechanisms Influence Bacterial Virulence and Survival

There are, however, several observations that do not fully fit with the K^+^ requirement models. Evidence from *S. aureus* suggests that K^+^ availability has little to do with KdpD/KdpE activity. Despite the fact that human blood is K^+^ rich, *S. aureus kdpD/kdpE* is still up-regulated and its deletion results in attenuated survival in that environment [18]. Additionally, *S. aureus kdpD/kdpE* transcription is altered not just during phagocytosis (when it is repressed); it is enhanced during biofilm formation and in response to microbicidal neutrophil extracts [20], [21], situations in which K^+^ is not necessarily limited. Although, it is important to remember that it is the KdpD/KdpE phosphorylation status that is ultimately important for activity rather than the transcription level of the genes themselves. Lastly, it has been suggested that the *S. aureus* Kdp-ATPase does not even function as a predominant K^+^ transporter [17]. It should be noted, however, that this suggestion was based on results from the deletion of only one KdpD/KdpE system when in fact some *S. aureus* strains possess two systems, with one located on the Type II SCCmec mobile genetic element [22]. Therefore, clarification of whether the strain used by Xue et al. (NCTC 8325) carries one or more functional Kdp operons would be pertinent.

### KdpE Is a Regulator of Diverse Virulence Loci

**Figure 1:**
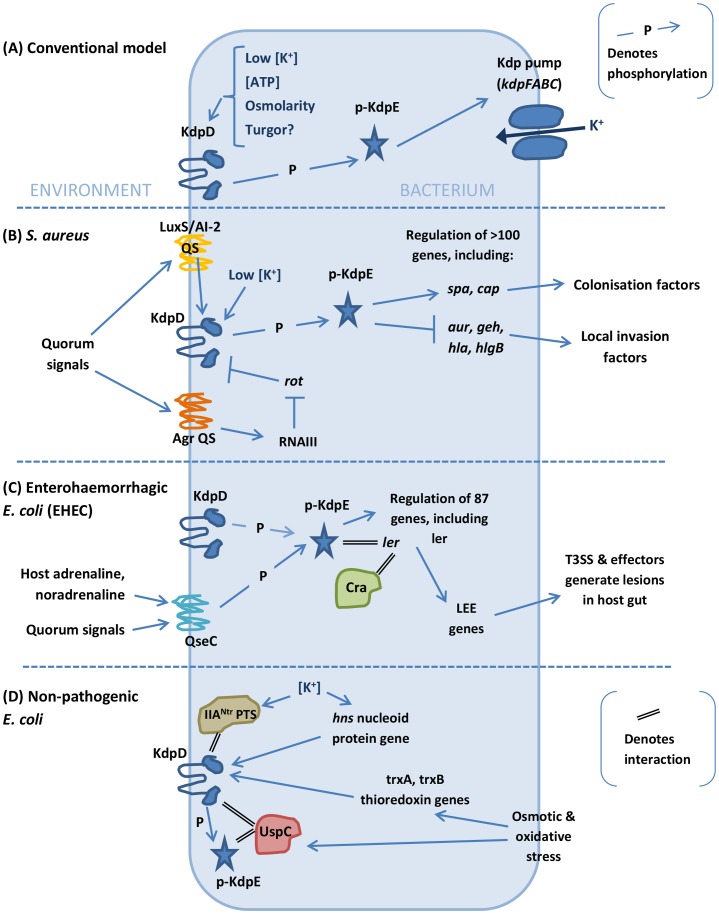
Schematic diagram of the varied inputs, accessory proteins, and regulatory effects of KdpD/KdpE. (A) The conventional model is that KdpD/KdpE stimulates transcription of the Kdp-ATPase in response to cytoplasmic ionic and ATP concentrations and possibly also turgor pressure [9]. (B) In *S. aureus* KdpD is affected directly or indirectly by QS systems, and KdpE regulates many downstream genes including virulence factors by directly binding to their promoters [17], [18]. (C) In EHEC, KdpE can also be activated by the QseC histidine kinase, which senses host adrenergic signals as well as bacterial quorum sensing (QS) signals [24]. In vitro its regulatory targets include the *ler* gene, which controls the “locus of enterocyte effacement" (LEE) genes. Under gluconeogenic conditions, KdpE interacts with Cra to optimally regulate *ler*; both proteins bind to the promoter, perhaps through bending of the DNA [25]. The downstream regulatory cascade is integral to lesion formation in the host gut [24], [25]. (D) Recently identified accessory components in nonpathogenic *E. coli* link the pathway to additional input stimuli or modulate KdpD activity [34], [37], [50].

As well as a regulator of the Kdp-ATPase, recent evidence supports the role of KdpE as a direct regulator of *S. aureus* virulence factors [17], [18]. KdpE has been shown to bind directly to the DNA promoters of a range of virulence genes and it has been demonstrated that deletion of *kdpD/kdpE* altered the level of transcription of over 100 genes, including (i) the surface protein gene *spa* (Staphylococcal protein A), (ii) the capsule synthesis gene *cap*, (iii) the alpha-toxin gene *hla*, (iv) the metalloproteinase aureolysin (*aur*), (v) the lipase gene *geh*, and (vi) the gamma-haemolysin gene *hlgB* [18]. Figure 1B depicts virulence-related regulatory targets of KdpD/KdpE in *S. aureus*. A positive regulatory effect was elicited upon the colonisation genes (*spa* and *cap*), and a negative regulatory effect was elicited upon the local invasion enzymes and toxin genes (*hla*, *aur*, *geh*, and *hlgB*) [17]. *S. aureus kdpD* is most highly transcribed at low external K^+^. This means that colonisation is enabled in response to low K^+^ (in the external environment, for example), and once in higher K^+^ conditions (in blood or cytoplasm), local pathogenesis is enabled. The *spa* gene has previously been shown to enhance virulence in mouse and macrophage models due to its antiphagocytic role [23].

Activation of virulence genes via KdpE has also been reported for Enterohaemorrhagic *E. coli* (EHEC) strain O157:H7 [24]. The adrenergic receptor QseC enables EHEC to sense host adrenaline and noradrenaline, as well as sensing bacterial signalling molecules (Figure 1C). This means EHEC can exploit host signals to regulate its metabolic, virulence, and stress response genes via a complex signalling cascade. In addition to its cognate RR, the QseC HK activates two additional RRs, one of which is KdpE [24]. In an in vitro system employing liposomes and purified proteins, over 80 QseC-regulated genes were found to be activated via KdpE. Amongst its targets were the type 3 secretion system (T3SS) and its effectors (via regulation of *ler*), which are necessary genes for “attachment and effacement" lesions of the intestinal epithelia (see Figure 1) [24]. These results show that KdpE has at least the potential to regulate critical virulence genes during infection. Further in vitro research has shown that KdpE directly interacts with a second transcription factor, the catabolite repressor/activator protein Cra, to regulate *ler* in a glucose-dependent manner [25]. Visualisation of infected HeLa cells revealed that deletion of *kdpE* resulted in slightly fewer lesion pedestals than wild-type, though the effect was more pronounced for the equivalent *cra* mutation. Importantly, however, in a double mutant, full complementation was achieved only when both proteins were expressed, indicating that both transcription factors are co-involved in regulating that aspect of EHEC virulence. Additional in vivo studies using a noncomplemented *kdpE* knockout strain and whole organism infection models would further help to dissect to what extent KdpE plays a direct role in EHEC virulence.

## Signal Integration via the Sensor Kinase KdpD and the Relevance for Host Infection

Rather than representing two distinct concepts, bacterial virulence and survival may be considered “two sides of the same coin." Genes that promote the survival of a pathogen in its host—whether via stress response or by direct regulation of virulence genes—may be classed as virulence factors. While EHEC and *S. aureus* provide examples for the direct regulation of virulence genes by KdpE, there remains a more general implication of the importance of KdpD/KdpE in virulence mediated by stress resistance.

### Stress Sensing and Resistance

There is a significant body of evidence supporting a link between KdpD/KdpE and the bacterial stress response, which in itself has clear relevance to survival within a host. With K^+^ concentration being an activating stimulus for Kdp-ATPase expression, it is understandable that osmotic stress (salt shock) can be linked to activity of KdpD/KdpE. However, studies in several bacteria also link *kdpD/kdpE* to resistance to oxidative and antimicrobial stresses specifically. Importantly, these bacteria are all either obligate or facultative intracellular pathogens. All have been reported in some instance to be able to resist host killing [26]–[28], and the majority are known to spend at least part of their lifecycle in the harsh phagosomal compartment—an acidic environment containing a barrage of hydrolytic enzymes, microbicidal agents, and reactive oxygen species (ROS) including hydrogen peroxide (H_2_O_2_). If KdpD/KdpE does mediate the sensing of such stresses encountered in the host, then the effect of knocking them out in those infection models would be explained. Table 2 summarises the experimental evidence where KdpD/KdpE has been implicated in stress resistance.

**Table 2:** Evidence that KdpD/KdpE plays a role in resisting stresses.

Bacterium	Link to Stress Resistance	References
*Salmonella typhimurium*	*kdpD* KO increases sensitivity to antimicrobial stress (polymyxin B), osmotic stress (NaCl), and oxidative stress (H_2_O_2_); the KO mutant elicits a 3-fold greater host response (production of nitric oxide) from macrophages	Alegado et al., 2011 [19]
*Francisella novicida*	Transposon insertion mutants in *kdpD* and *kdpE*, as well as *kdpA* and *kdpC*, are more sensitive than the wild-type strain to oxidative stress (H_2_O_2_ and paraquat)	Moule et al., 2010 [47]
*Yersinia pestis*	KdpD/KdpE KO results in hypersensitivity to crude neutrophil granule extracts and to five individual neutrophil microbicides; amongst a panel of deletion mutants the effect on antimicrobial peptide resistance is second only to that of PhoP/PhoQ	O'Loughlin et al., 2010 [3]
*Photorhabdus asymbiotica*	*P. asymbiotica* KdpD/KdpE enables *E. coli* to persist in phagocytes whereas the native KdpDE does not; the greatest amino acid divergence is in KdpD's Universal Stress Protein (Usp) domain, thought to be involved in stress sensing	Vlisidou et al., 2010; Heerman and Fuchs, 2008 [38], [40]
Enterohaemorrhagic *Escherichia coli* (EHEC)	KdpE is activated by the sensor kinase QseC, a receptor for adrenergic signals; adrenergic regulation of bacterial gene expression is an underlying mechanism of stress response and cellular survival	Hughes et al., 2009 [24]

Further evidence that KdpD can sense host-derived signals—including evidence for a role in stress response—was seen in *Bacillus cereus*, in which exposure to chitosan led to up-regulation of the *kdpABC* and *kdpD* genes [29]. Chitosan is an antibacterial polysaccharide, thought to induce loss of ions and cellular turgor by forming pores in bacterial membranes. This might suggest that the Kdp-ATPase was up-regulated in order to recover lost K^+^. Yet two observations suggest otherwise. One is that a deletion mutant of the Kdp-ATPase structural genes actually showed no impairment of growth in conditions of either K^+^-limitation or salt-stress, bringing into question the importance of Kdp for K^+^ regulation in *B. cereus*. The other is that exposure to benzalkonium chloride (a disinfectant that generates leakage of intracellular ions in much the same way as chitosan) did not have the same effect of up-regulating the *B. cereus kdp* genes [30]. Taken together this suggests that ion loss alone was not being sensed; rather, some other form of “antimicrobial stress" may have been. Indeed there is precedence for a TCS being directly activated by host-elicited antimicrobial peptides (AMPs) in the case of PhoP/PhoQ [31]. It is therefore tempting to speculate that the biologically derived antimicrobial chitosan may be sensed by KdpD in a manner akin to the sensing of small innate immune molecules by PhoP.

### Quorum Sensing

As discussed earlier, in various bacteria KdpD can integrate multiple stimuli including K^+^ concentration, osmolarity, possibly turgor pressure, and even host adrenergic signals. Importantly, in *S. aureus* KdpD/KdpE can also respond to population density. The two studies that showed direct regulation of *S. aureus* virulence factors also reported that KdpD/KdpE can integrate signals from two quorum sensing (QS) systems—the *luxS*/AI-2 system [18] and the Agr system [17]. Therefore, KdpE-regulated *S. aureus* virulence genes are expressed appropriately according to both K^+^ concentration (an indication of whether the bacterium is extra- or intracellular), and population density (indicating whether there are enough bacteria present to make a particular response appropriate, e.g. toxin production) [17]. As such, *S. aureus* uses KdpD/KdpE in an adaptive manner, utilizing its “traditional" sensor activity in an integrated fashion with input from QS systems.

### Accessory Components of KdpD

Accessory proteins (also known as auxiliary factors) are known to affect TCSs by (i) fine-tuning the ratio of the HK's kinase- to phosphatase-activity, (ii) linking the system to other regulatory networks, or (iii) enabling the sensing of additional stimuli [32], [33]. Table 3 summarises accessory proteins that have been identified for KdpD, and *E. coli* accessory factors are also shown in Figure 1D. It is not clear if there is any one specific region of the KdpD protein structure that is responsible for fine-tuning sensor kinase activity through accessory protein interactions. For example, it has been suggested that the IIA^Ntr^ protein (see below) interacts with the C-terminal transmitter domain of KdpD [34]. On the other hand, the N-terminus includes a conserved “KdpD" subdomain with a regulatory ATP-binding site [35], [36], and a universal stress protein (KdpD-Usp) subdomain, which is highly variable between species. The *E. coli* universal stress protein UspC is known to interact with the N-terminal Usp-domain thus altering its activity [37], although not through an influence on KdpD kinase activity.

**Table 3:** Identification of KdpD accessory components in *E. coli* and *M. tuberculosis*.

Bacterium	Component	Details	References
*Escherichia coli*	IIA^Ntr^ (enzyme IIA of the Ntr phospho-transferase system)	Interacts with KdpD, stimulates auto-kinase activity, and therefore Kdp pump expression; this activity is linked to glycolytic growth	Lüttmann et al., 2009; Njoroge et al., 2012 [25], [34]
	*trxA* (thioredoxin 1), *trxB* (thioredoxin reductase)	Thioredoxins control the bacterial stress response by maintaining a reducing environment in the cell, preventing ROS damage, and signalling osmotic stress and low pH; these genes exert effects upstream of KdpD, though the precise nature of the interaction is not known; KO of either gene results in reduced expression of the Kdp pump	Sardesai and Gowrishankar, 2001; Kumar et al., 2004; Zeller and Klug, 2006; Ehrt and Schnappinger, 2009 [50], [52]–[54]
	*hns* (H-NS nucleoid protein)	Exerts effects upstream of KdpD, though the precise mechanism of the interaction is not known; KO results in reduced expression of the Kdp pump	Sardesai and Gowrishankar, 2001 [50]
	UspC (Universal stress protein C)	Binds to the Usp sub-domain of KdpD under osmotic stress conditions, scaffolding the active KdpD/phospho-KdpE/DNA complex and stimulating expression of Kdp pump structural genes	Heerman et al., 2009 [37]
*Mycobacterium tuberculosis*	LprJ, LprF (membrane lipoproteins)	Either protein can interact specifically with KdpD (solely with the C-terminal region or while also forming a ternary complex with the N-terminal region); appear to be involved in the phospho-relay process; likely act as sensors or ligand-binding proteins for as-yet-unknown signals/ligands	Steyn et at., 2003; Buelow and Raivio, 2010 [33], [43]

### Is the Usp Domain of KdpD Involved in Stress Sensing?

A fundamental feature of Usp proteins is that they accumulate under stress conditions, and indeed UspC binds to the KdpD-Usp domain in response to osmotic stress, stimulating *kdpFABC* expression by acting as a scaffold between the active KdpD/phospho-KdpE/DNA complex [37]. This enables Kdp-ATPase expression at K^+^ levels at which it would normally be repressed. It has previously been suggested that the KdpD-Usp domain might be pivotal in the sensing of additional stimuli [19], [38]. UspA proteins protect *E. coli* against superoxide stress, and the UspA proteins of *Photorhabdus* species and *Yersinia enterocolitica* are thought to play a role in stress sensing when invading insect hosts [37], [39], [40]. *Photorhabdus asymbiotica* is a facultative intracellular pathogen of insects and humans. Interestingly, when *P. asymbiotica kdpD/kdpE* was expressed in a nonpathogenic *E. coli* strain, the presence of the transgenic TCS conferred an ability to the *E. coli* strain to persist within insect phagocytic cells [38]. Although further molecular experiments would be required in order to identify the precise mechanism behind the phenomenon, this observation does show that there are differences in the functioning of the KdpD/KdpE TCSs between the two species. The greatest evolutionary divergence of the *kdpD* and *kdpE* genes from *P. asymbiotica*, *E. coli*, and other bacterial species does indeed reside within the KdpD-Usp domain. Interestingly, domain swapping experiments revealed that this domain is also crucial for activation of KdpD. While the Usp-domain has a net-positive charged surface, integration of a net-negative charged surface was shown to block the activity of the protein. Therefore, this domain can not only act as an interaction surface for other proteins, but also appears important for internal (de)activation of KdpD [41]. It has been suggested that binding of other Usp proteins might either alter the secondary structure of the protein or perhaps facilitate activation by shaping a more positive surface at this position. The ability of KdpD/KdpE to sense and respond to stress may thus be mediated via this Usp-binding domain, and the variability of that domain between species might contribute to differences in bacterial virulence and lifestyle.

## Overview

A study of the TCSs of *M. tuberculosis* in 2003 found that deletion of *kdpD/kdpE* unexpectedly resulted in hypervirulence in mice [42]. Although no targets of KdpD/KdpE other than the Kdp-ATPase itself had been identified at the time, the result prompted the suggestion that KdpD/KdpE deletion had de-repressed virulence genes. It is possible that several mycobacterial lipoproteins identified in a study by Steyn et al. [43] might be targets of KdpD/KdpE regulation. Since then, a diverse set of observations in a range of species supports the role of KdpD/KdpE in promoting survival and virulence through various mechanisms distinct from K^+^ regulation. In most cases it is not yet clear exactly how KdpD/KdpE impacts on virulence—it is likely that the link to stress response aids bacterial persistence within host cells. KdpD/KdpE's role in virulence is likely to be highly variable across species and may ultimately be an indicator of species-specific adaptation.

In both Gram positive and Gram negative pathogens, KdpD/KdpE functions as a previously unrecognised adaptive TCS of stimuli during host infection, integrating cues from the host (phagocytosis, host-derived antimicrobials, and adrenergic hormones) in addition to bacterial quorum-sensing signals and the outside ionic concentrations. A recent review of “moonlighting" bacterial proteins focused upon glycolytic enzymes and molecular chaperones in a range of pathogens. Just as the evidence suggests for KdpD/KdpE, this review emphasized that key metabolic proteins and molecular chaperones, essential for dealing with the bacterium's response to stress, also have unexpected functions in virulence [44]. Like key metabolic enzymes and molecular chaperones, the KdpD/KdpE TCS usually plays a conserved role in maintaining normal cellular function. It seems that the conserved TCS KdpD/KdpE provides a further example of bacterial protein “moonlighting." It is suggested that proteins can display multifunctionality due to “biochemical" or “geographical" conditions: either through possessing additional biochemical reactions or by being located in a different cellular location (such as the pole of an asymmetric cell), or both [44]. There appear to be no reports of KdpE or KdpD being located anywhere other than the cytoplasm and cytoplasmic membrane, but evidence does suggest that the Usp domain is a prime candidate as an additional “biochemical" site. Data regarding the effects of KdpD/KdpE in pathogenesis are somewhat limited to date but come from a range of bacterial genera and experimental approaches. The evidence reveals a spectrum of Kdp function from a clearly homeostasis-based role to a highly pleiotropic one. Given its function in K^+^ homeostasis, the KdpD/KdpE TCS is extremely important for bacteria in challenging environments. We argue that it is therefore likely to be a focal point for selection in relation to survival during infection. It should be noted that no additional functions have yet been ascribed to KdpD/KdpE in nonpathogenic *E. coli*; accessory components have been identified that interact with *E. coli* KdpD, but these can be reasonably linked to K^+^ homeostasis. Examples include thioredoxins, which are integral in stress response pathways involving osmotic stress, the H-NS nucleoid protein, which is a transcriptional repressor that can respond to K^+^ concentration [45]; and the IIA^Ntr^ enzyme. This enzyme has been shown to connect K^+^ homeostasis with carbon starvation through direct interaction of nonphosphorylated IIA^Ntr^ with KdpD activating the kinase activity [46]. Nonpathogenic *E. coli* are not typically capable of surviving phagocytosis, and yet when expressing the KdpD/KdpE TCS from *P. asymbiotica*, *E. coli* cells did persist intracellularly [38] implying a fundamental difference between the abilities of the two systems to sense or respond to the phagocytic environment. The evidence from *F. novicida* can be adequately explained by a role in K^+^ regulation; mutants in the *kdpFABC* operon (as well as those in regulatory genes) were attenuated in the fruit fly model, which indicates that the Kdp-ATPase itself is important in this species [47]. Yet in *F. tularensis*, only the *kdpD* gene mutant was identified as attenuated in the mouse model; *kdpFABC* operon genes were apparently less critical than within the gene encoding the sensor kinase [48]. In EHEC, KdpE regulates K^+^ uptake and osmolarity alongside important virulence genes; the EHEC QseC HK, which activates KdpE, has even been investigated as a potential target for novel “anti-virulence" drugs [24], [49]. KdpE interacts with Cra, which itself coordinates the *E. coli* response to sugar availability, thereby linking metabolism to pathogenesis. It has been suggested that during its evolution EHEC has co-opted “established mechanisms for sensing the metabolites and stress cues in the environment, to induce virulence factors in a temporal and energy-efficient manner, culminating in disease" [25]. The clearest K^+^ independent role in virulence so far is in *S. aureus*, in which KdpD responds to population density and KdpE actually regulates over 100 genes.

## Concluding Remarks and Future Directions

In these cases where KdpD/KdpE has been shown to regulate additional virulence genes, it is not yet clear how this has evolved. For example, did the promiscuous regulation by KdpE evolve through changes in the KdpE DNA-binding domain, or has there been widespread selection for the evolution of KdpE operator sequence sites across a range of virulence loci? Furthermore, how taxonomically widespread is the expanded regulatory capacity of KdpE, and has the same mechanism occurred in each case? If specific virulence genes have evolved KdpE-responsive promoters, then we may expect to see similar virulence genes under the control of KdpD/KdpE in different pathogens by virtue of horizontal gene transfer. EHEC's T3SS and lesion-causing genes were acquired on a mobile genetic element, and it has been suggested that Cra and KdpE (originally regulators of nonpathogenic functions) have been “co-opted by a pathogen to regulate virulence factors encoded within a horizontally acquired [pathogenicity island]" [25].

**Figure 2:**
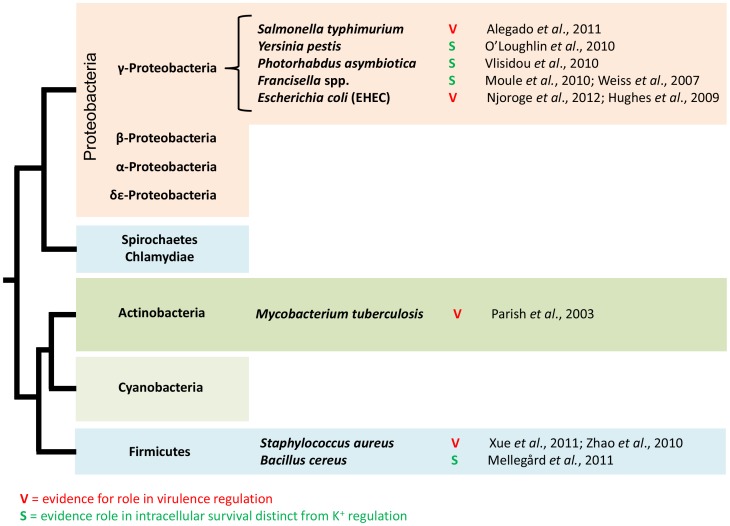
KdpD/KdpE virulence-related roles across bacterial taxa. Evidence supporting the role of KdpD/KdpE in virulence (V) or survival (S) is indicated across diverse bacterial species, all of which are capable of intracellular replication to some extent. The relevant references are also indicated. Phylogenetic relationships are as suggested by Battistuzzi et al. (2004) (not drawn to scale) [51].

KdpD/KdpE is positioned at a molecular hub that is fundamentally relevant to homeostasis during infection. This central position within regulatory networks controlling homeostasis, in conjunction with the radical stress associated with pathogenic host interactions, is likely to explain how KdpD/KdpE has evolved a role in pathogenicity in some taxa. As such, KdpD/KdpE is likely subjected to strong selection to acquire novel functions in mediating virulence pathways. We might expect that the selection pressures, or the novel functions that result from them, would be variable across different bacteria with different hosts and infection parameters. This taxa-specific evolutionary novelty is reflected in the phylogenetically diverse yet discontinuous nature of the data available so far. Figure 2 depicts the phylogenetic relationship between bacterial taxa where evidence supports a pleiotropic role of KdpD/KdpE. The majority of cases come from the well-studied gamma-Proteobacteria clade, but there is also evidence from the more distantly related Firmicutes and Actinobacteria. The common association of KdpD/KdpE within an operon containing the structural genes KdpABC suggests that a role in K^+^ homeostasis is ancestral and that KdpD/KdpE's adaptation for virulence or survival has arisen more recently and possibly multiple times. Whether K^+^ regulation by KdpD/KdpE pre-dates more specific host-responsive activities or whether these evolved in parallel is a key question with broad implications on the evolution of virulence. Ultimately, a detailed understanding (across a range of pathogenic and nonpathogenic bacteria) of KdpD/KdpE's connectivity within homeostatic response, stress response, and virulence pathways will be needed to address this question.

The perception that Kdp serves a general homeostatic function alone means that the Kdp regulatory network has not been specifically targeted in virulence studies. The studies discussed in this review, however, point to a clear need for more detailed experiments, with a systematic approach across multiple taxa. Specifically, useful tools would include (i) genetic knock-outs (plus/minus complementation) of KdpD and KdpE (individually and together) and the structural genes *kdpFABC* or (ii) combinations of transgenic and/or native TCSs and Kdp-ATPases from species observed to show different effects of KdpD/KdpE. These genetic tools could be used for transcriptomic analyses and in vivo studies. Transcriptomic analyses could identify networks of genes co-regulated in response to different challenges (K^+^ limitation, AMPs, oxidative or osmotic stress) or during infection of cells and whole organisms. In vivo studies in host organisms could examine the effects on acute virulence (measuring LD50, time to death, or host weight gain) or chronic persistence (how long bacteria can be retrieved). Ideally, it would be informative to re-investigate the species discussed in this review—and new species—using a coordinated and comparable experimental approach. To contrast with the data available so far, it would be interesting to investigate species that cannot replicate intracellularly such as *Pseudomonas* spp. (gamma-Proteobacteria) or *B. cereus* (Firmicutes). Studies involving closely related species with diverse lifestylessuch as the *Burkholderia*, which range from environmental organisms to obligate and opportunistic pathogens with varied host ranges—might be useful for dissecting the effects of host and lifestyle on KdpD/KdpE function.

We predict that KdpD/KdpE will continue to be demonstrated to function in K^+^ homeostasis and virulence (directly or via stress response etc.) to different extents in different species. These differences are likely to correlate with whether the species in question leads a predominantly pathogenic or more environmentally based lifestyle. In the former case, the type of host response a species encounters is also likely to be important. Finally, studies of TCSs such as PhoP/PhoQ, Cra, and KdpD/KdpE suggest that other central homeostasis bacterial regulators will also prove to have various adaptive roles in virulence. The application of sophisticated systems-level studies across diverse bacterial pathogens may help to clarify this phenomenon and take us closer to understanding the regulatory breadth and complexity of pathogenesis.
